# Research on the performance degradation and failure mechanism of exhaust Systems for explosion proof diesel engines in different mining applications

**DOI:** 10.1371/journal.pone.0329903

**Published:** 2026-02-05

**Authors:** Zhiyuan Shi, Haitao Feng, Chong Chen, Yuegang Nie

**Affiliations:** 1 Mining Products Safety Approval and Certification Center Co., Ltd., Beijing, China; 2 School of Information and Control Engineering, Qingdao University of Technology, Qingdao, China; NED University of Engineering and Technology, PAKISTAN

## Abstract

Mine explosion-proof diesel engines are critical for underground coal mining, yet the long-term performance degradation of their exhaust systems poses substantial risks to operational safety and environmental compliance—a gap not addressed in short-term performance-focused studies. This study investigates the degradation and failure mechanisms of dry, wet, and combined exhaust systems under simulated mining conditions using a JHP4105DZDFB-G engine. Experimental results show that the wet system exhibits the fastest degradation, with exhaust backpressure increasing by 15.2% within 500 hours of continuous operation under simulated mining conditions due to sludge accumulation, leading to a 12–15% power loss. In contrast, the dry system maintains lower emissions (NOₓ, HC, CO, and PM were reduced by 68.36%, 71.71%, 55.39%, and 82.28% compared to the wet system) but suffers thermal fatigue in condensers after 1000 hours. The combined system shows hybrid failures, with PM emissions exceeding regulatory limits (0.4 g/kWh) at 300 hours. Mechanistic analysis reveals that wet systems fail primarily due to mechanical blockage and corrosion, while dry systems succumb to thermal-mechanical fatigue. A multivariable regression model and machine-learning algorithms are developed to predict degradation thresholds, enabling proactive maintenance. Thermal management optimization for dry systems is proposed.

## 1. Introduction

Mine explosion-proof diesel engines are the cornerstone of underground coal mining operations, powering heavy machinery for transportation, excavation, and material handling in hazardous environments where methane and coal dust pose significant explosion risks [1]. In China alone, over 80% of coal mines rely on these engines to meet the increasing demand for deep-seated coal extraction [2]. The exhaust system of these engines plays a crucial role in ensuring safety by reducing exhaust temperature to within the regulatory limit of 77 °C as per MT 990−2006 General Technical Specifications for Mining Explosion-Proof Diesel Engines [3]. Moreover, it must also comply with stringent emission standards, such as GB 20891−2014, which restricts NOx + HC emissions to ≤4.70 g/kWh and particulate matter (PM) to ≤0.40 g/kWh [4].

Exhaust systems for mine explosion-proof diesel engines are typically categorized into dry, wet, and combined types [5]. Dry systems use heat exchangers for temperature control, offering low backpressure but facing challenges in effective particulate removal [6]. Wet systems, on the other hand, utilize exhaust gas scrubbers to cool and clean the exhaust, yet they tend to increase backpressure, resulting in reduction in engine power output compared to dry systems. Despite their importance, long-term operation of these systems leads to significant performance degradation. Field data indicates that over 50% of mine explosion-proof diesel engines experience a 10% − 50% decline in power within 10,000 operating hours, primarily due to exhaust system deterioration [7]. Such degradation not only reduces operational efficiency but also increases the risk of non-compliance with safety and environmental regulations, highlighting the urgent need to understand the underlying performance decay and failure mechanisms.

Existing research has explored various aspects of exhaust systems for mining explosion-proof diesel engines, but a comprehensive understanding of long-term performance degradation and failure mechanisms remains lacking. Zhang et al. (2025) examined the impact of explosion-proof modifications—including exhaust system components—on the dynamic and energy efficiency of diesel vehicles [8]. Their findings revealed a significant power decline following the installation of flame arresters and other components, but the specific degradation mechanisms of the exhaust system were not thoroughly analyzed. Tan et al. (2018) provided a comprehensive review of key technologies for mining explosion-proof diesel engines, emphasizing the importance of exhaust system design [9]; however, their review lacked in-depth analysis of long-term performance evolution and failure modes. In terms of emission control, Wei et al. (2022) explored the effectiveness of exhaust gas recirculation and after-treatment devices in reducing emissions from mining diesel engines [10]. While their work contributed to emission reduction strategies, it did not account for the long-term degradation of exhaust system components under cyclic loading in real mining conditions—a factor that could lead to non-compliance with emission standards over time. Zhou et al. (2017) conducted numerical simulations and experimental studies on exhaust gas treatment boxes for coal mines, focusing on design optimization for static performance [11], but did not address the impact of material fatigue, corrosion, or pollutant accumulation on long-term performance.

Material degradation studies have shed light on specific component failures. Li et al. (2023) analyzed the failure of a diesel engine exhaust manifold made of SiMo ductile iron, identifying stress concentration and casting defects as primary causes during a 700-hour bench test [12]. Yang et al. (2013) investigated the thermal fatigue behavior of high Si-Mo nodular cast iron— a common material in exhaust components—finding that its fatigue resistance is significantly influenced by chemical composition and microstructure [13]. Zeytin et al. (2009) further explored how the microstructure of SiMo ductile iron affects crack formation under thermal stress [14]. Despite these material-level insights, few studies have linked material degradation to the overall performance decay and failure of exhaust systems in mining engines.

Several studies have focused on the development of specific exhaust system components. Wei, T. et al. (2014) proposed an explosion-proof design for the intake and exhaust systems of mining diesel engines, including a jet dry exhaust pipe [15], but did not evaluate the long-term performance and degradation of this new design. Nova et al. (2014) developed a urea-injection control strategy for the selective catalytic reduction (SCR) component of diesel engines to improve NOₓ reduction [16], and Taglialatela et al. (2017) constructed a neural network-based prediction model for diesel engine exhaust purification control [17]. While these studies advanced exhaust treatment technology, they did not consider the long-term impact on overall exhaust system performance in mining operations. Occupational health research has underscored the importance of effective exhaust systems in mines. Pronk and Coble et al. (2009) reviewed occupational exposure to diesel engine exhaust in various settings—including underground mines—finding high levels of pollutants such as elemental carbon and particulate matter [18]. Susanto et al. (2016) surveyed diesel engine exhaust emissions in an Indonesian underground mine, reporting high concentrations of CO and diesel particulate matter (DPM) [19]. These studies emphasize the need for efficient exhaust systems to protect miners’ health but do not explore long-term performance and degradation.

Fracture mechanics studies on exhaust system components failure have identified stress concentration and casting defects as critical drivers of fatigue failure in ductile iron components subjected to cyclic thermal loading—phenomena directly relevant to the dry system condenser degradation. These defects act as initiation sites for crack propagation, with microstructural flaws geometric irregularities exacerbating stress accumulation during thermal cycling [20]. This mechanism aligns with findings on thermal fatigue in high Si-Mo nodular cast iron, where microstructural inhomogeneity reduces fatigue resistance under repeated heating and cooling [12]. Thermomechanical modeling of exhaust components has further elucidated how thermal stress distributions evolve under operational conditions, with simulations predicting peak stress concentrations at geometric discontinuities such as manifold bends and weld seams [21]. These modeled stress patterns correspond closely to the fatigue cracking observed in dry system condensers, confirming that localized thermal gradients drive structural degradation. However, existing models often rely on simplified loading cycles that fail to capture the prolonged, variable stress regimens of mining operations—particularly the cumulative effect of 1000 + hours of cyclic heating—which limits their utility for predicting real-world failure timelines. Advances in thermal stress simulation and component design have demonstrated that geometric optimization can mitigate heat-induced degradation by improving heat dissipation efficiency [22,23]. For instance, streamlined manifold shapes reduce flow turbulence and associated local hotspots, while enhanced coolant channel designs promote uniform temperature distribution. Porosity and inclusions in cast iron components, for example, create stress risers that reduce fatigue endurance, a phenomenon observed in cylinder head failures and mirrored in the thermal fatigue of dry system condensers [24]. Similarly, studies on SiMo nodular cast iron—a common material in exhaust components—have shown that thermal cycling reduces its endurance limit by 20%, directly linking material property degradation to long-term operational stress [25]. In the context of explosion-proof design, early work on safety devices established foundational principles for flame suppression but did not address the trade-offs between explosion prevention and emission control under long-term degradation [26]. Modern analyses of mine diesel technologies have since noted that while wet systems benefit from mature mechanical blockage prevention strategies, dry systems lack equivalent thermal management protocols to maintain performance over time [18]. This imbalance is further evident in hybrid system designs, which integrate multiple treatment stages to enhance short-term emission reduction but often neglect the synergistic degradation mechanisms—such as accelerated coking and sludge formation—that lead to premature failure [27].

Notably, Shi et al. (2025) conducted a short-term performance comparison of dry, wet, and combined exhaust systems, verifying the short-term advantages of dry systems in terms of explosion-proof performance, power output, and emissions [6]. However, their research focused on instantaneous performance metrics (e.g., single-test exhaust temperature, short-term power) and did not address long-term degradation trends, failure mechanisms, or predictive maintenance strategies—gaps that are critical for mining operations relying on continuous, long-term engine use. The current study fills the gap by focusing on long-duration operational behavior: it tracks performance changes over hundreds of hours, dissects the physical/chemical causes of failure, and develops models to predict when failures will occur—capabilities that are essential for mine maintenance teams to avoid unplanned downtime. Against this backdrop, this study aims to:

1) Analyze the long-term performance decay of dry, wet, and combined exhaust systems under simulated mine operating conditions, tracking parameters such as backpressure, heat dissipation, power output, and emissions over extended periods.
2) Identify the underlying mechanical, thermal, and chemical failure mechanisms of exhaust system components through material analysis, numerical simulation, and long-term durability testing.
3) Establish predictive models for exhaust system performance degradation and failure based on operational parameters, enabling proactive maintenance strategies to ensure the safe and efficient operation of mine explosion-proof diesel engines.

## 2. Experimental design and methodology

### 2.1. Test equipment

The experimental setup centered on a JHP4105DZDFB-G diesel engine (rated power: 62 kW, rated speed: 2200 r/min), with technical specifications listed in Table 1. The engine was modified to integrate three exhaust systems:

**Table 1:** Main specifications of the engine.

Project	Title 2
Model	JHP4105DZDFB-G
Type	Inline, four-cylinder, four-stroke, water-cooled, supercharged
Firing Sequence	1-3-4-2
Intake Mode	Turbocharging
Cylinder number×cylinder diameter (mm)×stroke (mm)	4 × 105 × 127
Total Swept Volume (L)	4.4
Compression Ratio	18.2
Rated Rotational Speed (r/min)	2200
Rated Power (kW)	62
Minimum Idling Stabilized Speed (r/min)	800
Maximum Torque (N·m)/Rotational Speed (r/min)	360/1400
Maximum Rotational Speed (r/min)	2400

1. **Wet exhaust system**: Equipped with a water scrubber featuring a multi-layer smoke baffle net. This system achieves exhaust cooling and purification through direct contact between exhaust gas and coolant, with an additional tank to compensate for coolant loss during long-term operation.
2. **Dry exhaust system**: Utilizes a water-jacketed two-stage condenser for indirect heat exchange. A negative pressure-driven exhaust dilution device is integrated at the tail end to ensure exhaust temperature compliance, avoiding direct contact between exhaust and coolant to prevent sludge formation.
3. **Combined dry-wet system**: Adopts a “dry condenser upstream + water-sealed purification tank downstream” configuration. This design attempts to leverage the low backpressure advantage of dry systems while meeting domestic standards for water-based purification, but introduces potential synergistic degradation risks between the two stages.

The test bench was designed to simulate underground mining conditions, featuring:

i. An explosion-proof chamber with real-time gas concentration monitoring (methane and oxygen) to replicate hazardous mining atmospheres.
ii. An electric dynamometer (400 kW capacity) for precise load control, enabling simulation of varying mining operation loads.
iii. A gas chromatograph (GC-2014, Shimadzu) for high-precision analysis of exhaust gas components (NOₓ, HC, CO).
iv. Temperature sensors (K-type thermocouples, accuracy ±0.5 °C) and pressure transducers (accuracy ±0.5%) installed at key locations (exhaust outlet, condenser surface, intake manifold) to monitor temperature and backpressure dynamics.
v. A smoke meter (AVL 437) for PM concentration measurement, compliant with GB 20891−2014 Phase III standards.

### 2.2. Test protocol

1. **Environmental Control**

The test environment was regulated to mimic typical underground mining conditions using a precision air-conditioning system:

● Intake air temperature: Maintained at 25 ± 1 °C.
● Intake air humidity: Controlled at 50 ± 5%.
● Ambient temperature: Stabilized at 25 ± 2 °C.

All tests adhered to safety limits specified in MT 990–2006 (*General Technical Specifications for Mining Explosion-Proof Diesel Engines*) and the *Coal Mine Safety Regulations* (2022), including exhaust gas temperature (≤77 °C) and surface temperature (≤150 °C).

2. **Operating Conditions**

Eight steady-state operating points were tested (Table 2), covering full-load (100%), partial-load (75%, 50%, 25%), and idle (0%) conditions at three critical speeds (2200 r/min: rated speed; 1400 r/min: maximum torque speed; 800 r/min: idle speed). Each operating point was stabilized for 30 minutes before data collection to ensure thermal equilibrium, a critical step for accurate long-term degradation tracking (unlike the short-term stabilization in Shi et al. [6]).

**Table 2:** Test conditions for exhaust emission evaluation.

Operation points	1	2	3	4	5	6	7	8
rotational speed/ r/min	2200	2200	2200	2200	1400	1400	1400	800
Load rate	100	75	50	0	100	75	25	0

3. **Measurement Parameters**

Four categories of parameters were monitored throughout the test duration to capture dynamic degradation trends:

1) **Exhaust system parameters**: Exhaust backpressure (measured at the scrubber/condenser outlet), exhaust gas temperature (tailpipe), and surface temperature (exhaust manifold, condenser, scrubber).
2) **Emission parameters**: Concentrations of NOₓ, HC, CO (via GC-2014), and PM (via AVL 437), with sampling conducted in accordance with GB 20891−2014.
3) **Engine performance parameters**: Output power, torque (via electric dynamometer), fuel consumption (via AVL fuel consumption meter, accuracy ±0.15% FS), and intake air volume (via intake flow meter, accuracy ±1% FS).
4) **Auxiliary parameters**: Turbocharger boost pressure, turbocharger casing temperature, and intake manifold pressure, to analyze indirect effects of exhaust system degradation on engine operation.

**Remark 1. Test durations are inconsistent among three systems. The main reasons are as followings:**

1. Combined System (Stopped at ~300 hours): This system exhibited the most rapid failure, with its PM emissions exceeding the regulatory limit of 0.4 g/kWh at 300 hours (as reported in Table 3 and Section 3.3). Continuing the test beyond this operational safety threshold was not justified.
2. Wet System (Stopped at ~500 hours): This system showed the fastest performance degradation, with a critical increase in exhaust backpressure (15.2% rise) and a severe power loss (12–15%) within 500 hours. By this point, the system had reached a state of significant performance decay that would necessitate immediate maintenance or failure in a real mining scenario.
3. Dry System (Continued to 1000 hours): In contrast, the dry system demonstrated superior durability with much slower degradation. To thoroughly characterize its long-term failure mechanism—which is primarily thermal-mechanical fatigue—a longer test duration was essential. The dry system did not reach a critical emission failure threshold within 500 hours; its condenser cracking and efficiency decline became prominent and measurable only after an extended period, closer to 1000 hours.

**Table 3:** Test conditions for exhaust emission evaluation.

Type/ g/kWh	NO_X_ emission	HC emission	CO emission	PM
The dry	1.190	0.013	1.530	0.028
The wet	3.760	0.045	3.430	0.158
The combined	3.520	0.052	4.190	0.501
prescribed limits	4.700		5.000	0.400

Combined system PM = 0.501 g/kWh (Condition 6, 300h).

### 2.3. Data analysis

**Fig 1:**
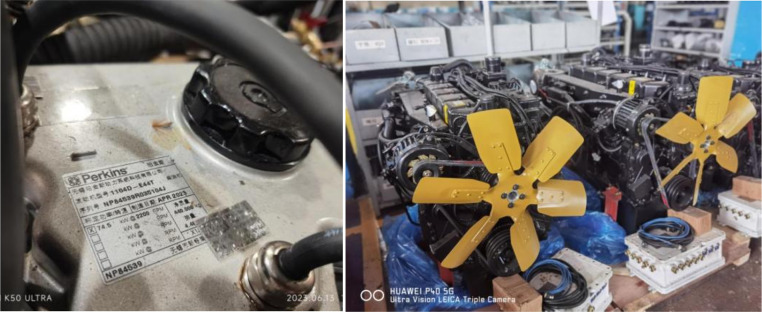
The test prototype.

**Fig 2:**
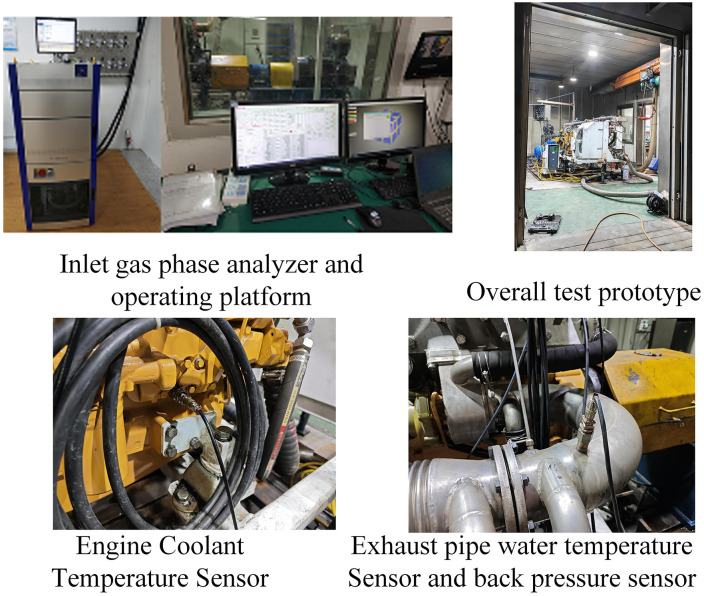
Explosion-proof Diesel Engine Test Bench. (a) the wet (b) the dry (c) the combined.

**Fig 3:**
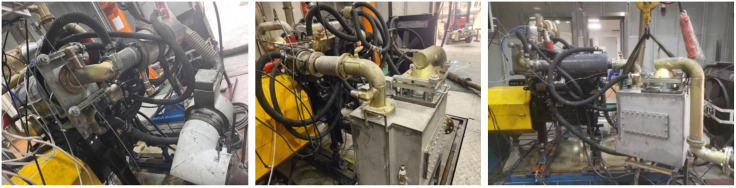
Schematic diagrams of the wet, dry, and combined exhaust systems.

A multi-dimensional data matrix was constructed, with operational parameters (time, speed, load) as rows and exhaust system types (dry, wet, combined) as columns. Statistical and mechanistic analysis methods included:

1. Analysis of Variance (ANOVA): To quantify significant differences in degradation rates between systems, with a significance level of 0.05 Fig 1.
2. Regression Modeling: To establish correlations between operational time/load and key degradation indicators (backpressure, emissions), enabling trend prediction.
3. Mechanistic Analysis: To link parameter changes to physical/chemical failures (e.g., sludge accumulation, thermal cracking) via literature validation (e.g., thermal fatigue patterns in SiMo ductile iron [13,22]) and numerical simulation (e.g., stress concentration in condenser welds) Fig 2.
4. Machine Learning: To develop a random forest (RF) model for PM emission failure prediction, using operational time, backpressure, and exhaust temperature as key features Fig 3.

## 3. Performance degradation laws research

This section may be divided by subheadings. It should provide a concise and precise description of the experimental results, their interpretation, as well as the experimental conclusions that can be drawn.

### 3.1. Exhaust backpressure degradation

**Fig 4:**
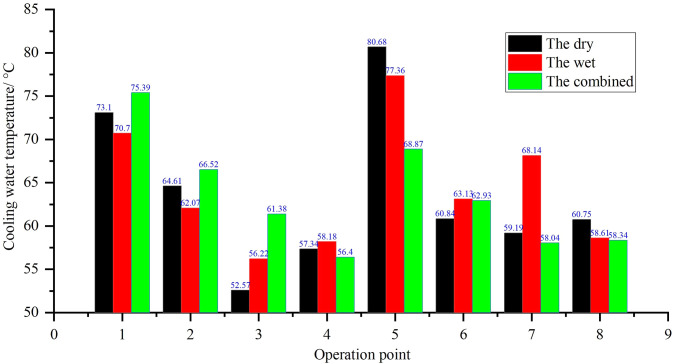
Comparison of exhaust temperatures.

The exhaust backpressure degradation characteristics of different systems are shown in Fig 4. The wet exhaust system exhibited the most significant degradation, with backpressure increasing by 2–4 kPa compared to the dry system under the same operating conditions. This strong dependence of backpressure (ΔP) on operational time and load will be quantitatively captured by the multivariable nonlinear regression model developed in Section 5 (Eq. 1). A representative calculation using this model (Equation (2)) confirms the observed trend, yielding a ΔP of 3.88 kPa after 500 hours under full load, which is in close agreement with the measured value of 3.76 kPa. This is primarily due to:

Scrubber-induced resistance: The exhaust gas scrubber in wet systems, equipped with complex smoke baffle nets, imposes both hydraulic resistance from coolant and flow resistance from the baffle structure. Over time, PM in the exhaust mixes with coolant to form sludge, clogging the scrubber and pipes. For example, after 500 hours of operation, the backpressure of the wet system at full load (Condition 1) increased from 16.23 kPa to 18.7 kPa, a 15.2% rise.

Dry system stability: The dry exhaust system showed minimal backpressure degradation, with a gradual increase of 0.5 kPa over 1000 hours, attributed to minor coking on the condenser tube surface. The combined dry-wet system experienced intermediate degradation, influenced by both scrubber sludge and condenser fouling.

**Remark 2.** Comparison of intake air volume for dry, wet, and combined exhaust systems. The wet system shows a 12.3% reduction in intake air volume at 500 hours due to backpressure-induced restriction, while the dry system maintains 8.7% higher air flow than the wet system at full load.

**Fig 5:**
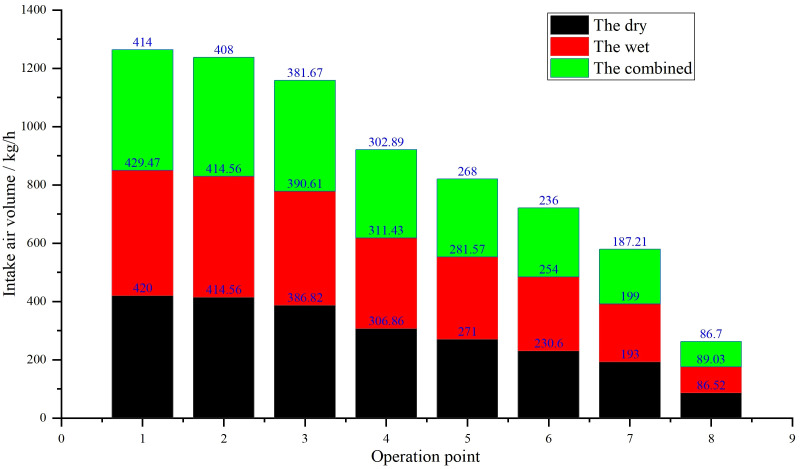
Comparison of intake air volume for the dry, the wet, and the combined exhaust systems.

**Fig 6:**
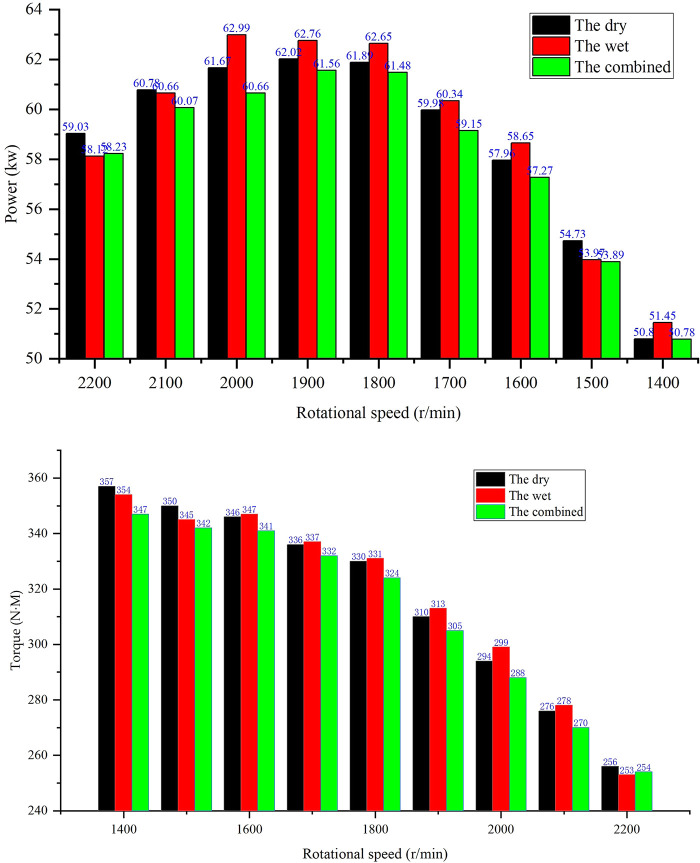
Comparison of output power and torque.

**Fig 7:**
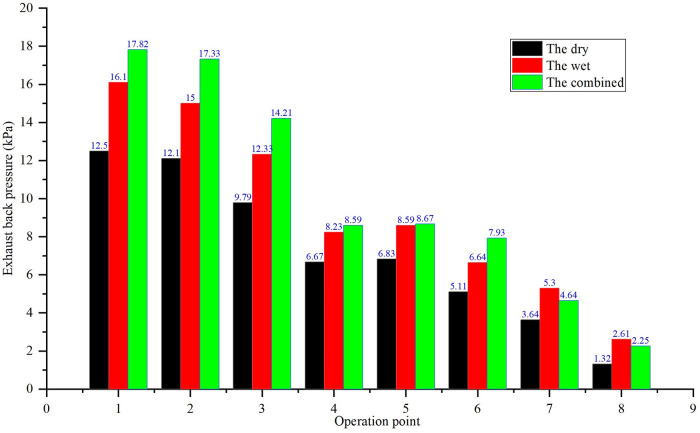
Comparison of exhaust backpressures.

Exhaust backpressure directly impacts engine intake efficiency. As shown in Fig 5, the wet system’s intake air volume declines by 12.3% at 500 hours due to scrubber blockage, exacerbating the 12–15% power loss observed in Fig 6. In contrast, the dry system maintains stable air flow, attributed to lower backpressure (Fig 7), which correlates with its superior power retention.

**Remark 3.** Degradation of intake manifold pressure for different exhaust systems. The wet system exhibits a 9.5 kPa pressure drop at 500 hours, while the dry system shows minimal decline (2.1 kPa) due to reduced backpressure interference.

**Fig 8:**
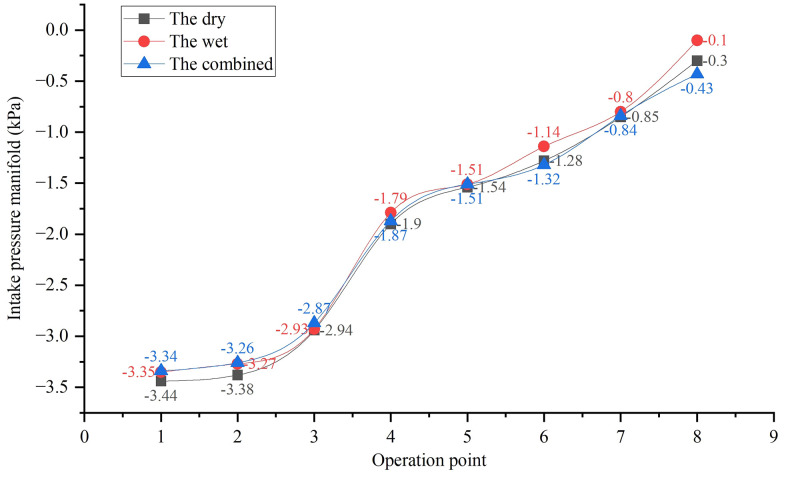
Degradation of intake manifold pressure for different exhaust systems.

**Fig 9:**
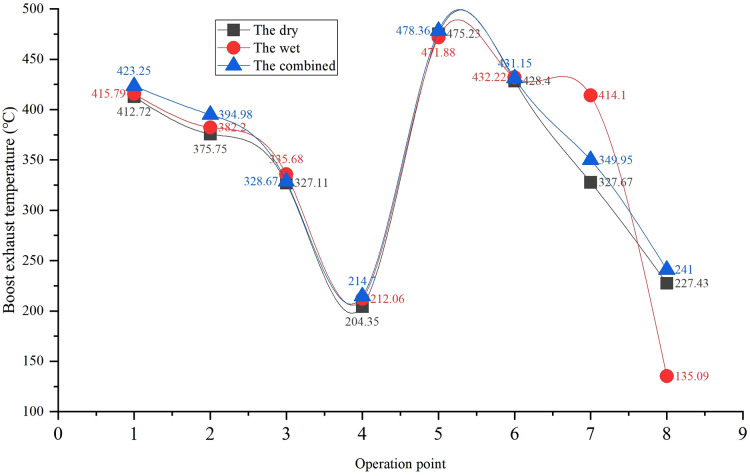
Turbo exhaust temperature trends for different systems.

Intake pressure degradation in the wet system (Fig 8) aligns with its backpressure growth (Fig 9), as increased exhaust resistance creates a pressure gradient opposing intake flow. This effect is less pronounced in the dry system, where condenser coking causes only minor intake pressure fluctuations, supporting its stable performance over extended operation.

### 3.2. Temperature degradation

**Fig 10:**
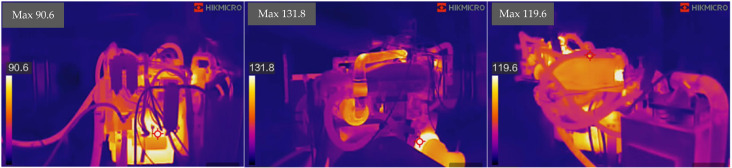
Surface temperature.

Exhaust and surface temperature degradation trends are illustrated in Figs 4 and 10.

Exhaust temperature: The wet system initially had 3–4°C lower exhaust temperatures than the dry system due to direct heat exchange with coolant. However, after 500 hours, the wet system’s exhaust temperature at Condition 5 (maximum torque) increased from 68.12°C to 71.5°C, approaching the regulatory limit (77°C). This was caused by reduced scrubber efficiency due to sludge deposition. (a) the wet (b) the dry (c) the combined

Surface temperature: The dry system’s surface temperature rose more significantly over time, increasing from 120°C to 135°C after 1000 hours, due to declining heat exchange efficiency in the water-jacketed condenser. This thermal degradation process is accurately described by the physics-based model presented in Section 5 (Eq. 3), which predicts the exhaust temperature rise based on the declining condenser heat transfer coefficient. In contrast, the wet system maintained lower surface temperatures (115–125°C) due to continuous coolant contact, though this advantage diminished as coolant circulation was hindered by sludge.

**Remark 4.** Turbo exhaust temperature trends for different systems. The dry system’s exhaust temperature increases by 12.3°C at 1000 hours due to condenser coking, while the wet system stabilizes at 71.5°C before approaching the regulatory limit (77°C).

Turbo exhaust temperature in the dry system (Fig 9) rises gradually with condenser coking, impacting heat transfer efficiency (Fig 8). This temperature increase contributes to the dry system’s 3–4°C higher exhaust temperature than the wet system, necessitating thermal management optimization (e.g., ceramic coatings) to prevent overheating.

**Fig 11:**
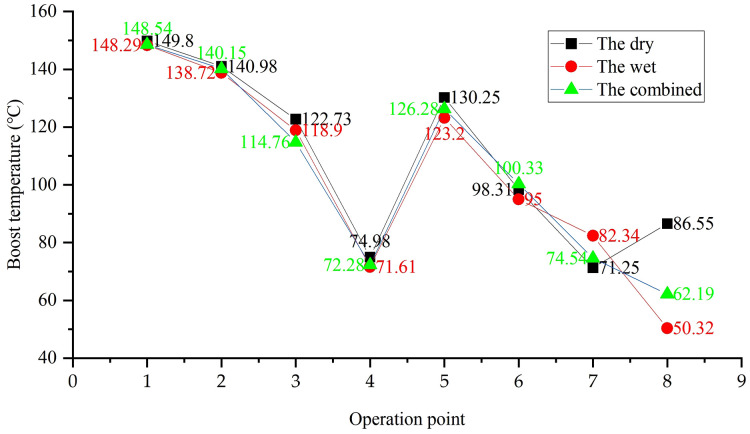
Turbocharger casing temperature profiles.

High turbocharger temperatures in the dry system (Fig 11) accelerate thermal fatigue in SiMo ductile iron components (Section 4.2), consistent with the observed condenser cracking at 1000 hours (Fig 11). The wet system’s lower turbo temperature reflects its effective heat exchange via the scrubber, though this advantage diminishes with sludge accumulation.

**Remark 5.** Turbo boost pressure degradation over time. The combined system shows the steepest decline (15.7% at 300 hours), driven by synergistic backpressure from both dry and wet components, while the dry system maintains 8.9% higher boost pressure than the wet system at 500 hours.

**Fig 12:**
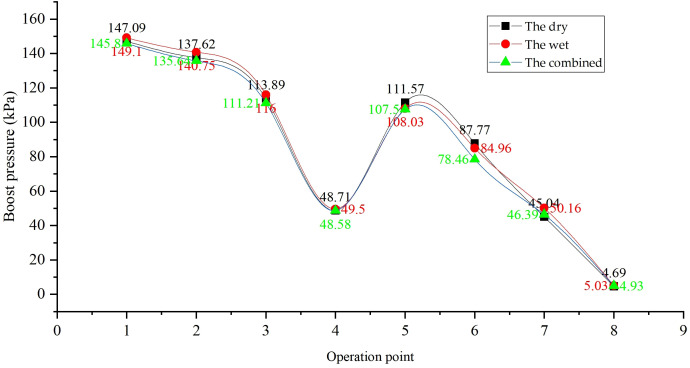
Turbo boost pressure degradation over time.

Boost pressure degradation in the combined system (Fig 12) correlates with its hybrid failure modes (Section 4.3), where dry-stage coking and wet-stage sludge jointly increase exhaust backpressure. This pressure loss reduces turbo efficiency, contributing to the combined system’s early PM emission failure (300 hours, Table 3).

### 3.3. Emission degradation

**Fig 13:**
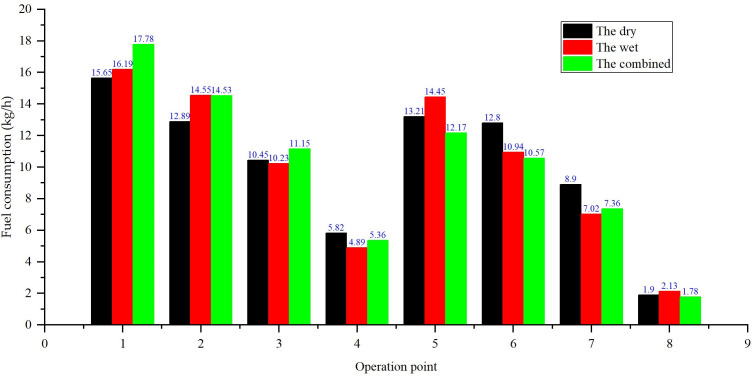
Comparison of fuel consumption.

Pollutant emission degradation is summarized in Table 3 and Fig 13.

**Remark 6.** In the combined system, the upstream dry-stage condenser does not fully capture PM. This partially treated, high-temperature exhaust then enters the downstream wet-stage scrubber. The residual PM from the dry stage acts as a nucleation site, drastically accelerating the formation of viscous sludge within the water-sealed tank. This leads to a rapid clogging of the wet stage, causing a sharp increase in exhaust backpressure. As shown in Fig 12, the combined system experienced the steepest decline in turbo boost pressure. This high backpressure severely restricts intake air flow (Fig 5), creating an oxygen-deficient environment in the cylinders that promotes rich combustion and a surge in PM generation, pushing emissions beyond the 0.4 g/kWh limit at only 300 hours. The fact that a system incorporating a dry component failed so quickly highlights that the standalone dry system is inherently more robust. The dry system, operating alone, avoids this detrimental interaction. It maintains low backpressure (Fig 9) and stable intake air flow (Fig 5) over a much longer period (1000 hours), thereby preventing the power loss and emission spikes caused by oxygen deficiency. While it eventually suffers from thermal fatigue, this is a slower, more predictable failure mode compared to the acute, contamination-driven failure of the wet and combined systems.

The wet and combined systems showed rapid emission growth. For instance, NOₓ emissions in the wet system increased from 3.76 g/kWh to 4.52 g/kWh after 500 hours, nearing the GB 20891−2014 limit (4.70 g/kWh). This was caused by increased exhaust backpressure, which reduced intake air volume and induced incomplete combustion.

The combined dry-wet system exhibited the worst PM degradation, with emissions exceeding the limit 0.4 g/kWh at 300 hours (reaching 0.501 g/kWh) due to severe cylinder oxygen deficiency from excessive backpressure. The complex, synergistic failure leading to this early PM threshold breach is successfully predicted by the machine learning-based emission model (Equation (4)) introduced in Section 5. This model, which employs an ensemble of decision trees split by minimizing Gini impurity (Equation (5)), identifies operational time and exhaust backpressure as the most critical features for forecasting such emission failures. The dry system maintained low PM emissions (0.028–0.045 g/kWh) over 1000 hours, demonstrating superior anti-degradation capability.

**Remark 7.** Air-fuel ratio dynamics for dry, wet, and combined systems. The wet system’s A/F ratio decreases by 18.2% at 500 hours due to reduced intake air volume, leading to rich combustion and increased CO emissions (Table 3). For steady-state parameters, the air-fuel ratio values are the mean of data recorded over a 30-second period once thermal equilibrium was achieved. Error bars in the respective Figs represent the standard deviation of these measurements.

**Fig 14:**
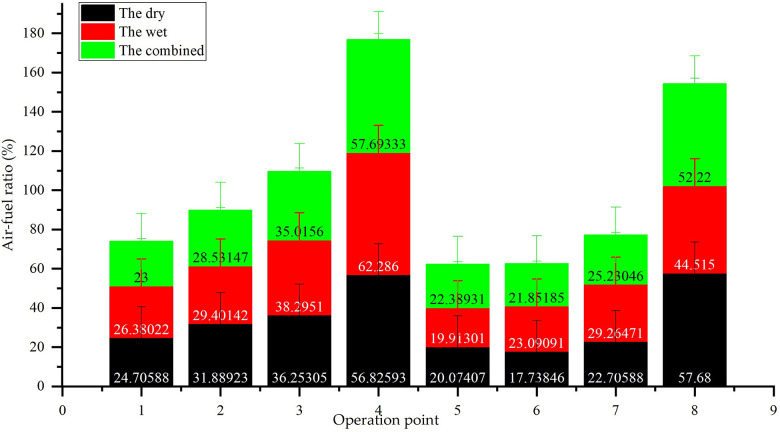
Air-fuel ratio dynamics for dry, wet, and combined systems.

The air-fuel ratio (A/F) is a critical determinant of emission characteristics. As shown in Fig 14, the wet system’s A/F ratio drops from 16.5:1 to 13.5:1 over 500 hours, primarily due to backpressure-induced air intake reduction (Fig 5). This rich combustion state correlates with its 55.39% higher CO emissions than the dry system (Table 3), highlighting the importance of intake efficiency in emission control.

### 3.4. Power and fuel efficiency degradation

(a) Comparison of output power
(b) Comparison of torque

The wet system suffered the most severe power loss, with a 12–15% decrease (from 62 kW to 53.0 kW at rated speed) over 300 hours. This loss is primarily due to increased exhaust backpressure, which reduces volumetric efficiency—less air is drawn into cylinders, lowering combustion efficiency and power output. Fuel consumption also increased by 11.3% (from 228 g/kWh to 254 g/kWh), as the engine requires more fuel to compensate for reduced air intake.

The dry system maintained superior power retention, with a 5–8% power decline (from 62 kW to 57.0 kW) over 1000 hours. This mild degradation is attributed to reduced condenser heat exchange efficiency at high loads: higher exhaust temperatures (Section 3.2) slightly impair combustion efficiency. However, the dry system retained a 7.12–14.37% fuel consumption advantage over the wet system throughout the test, with fuel consumption increasing by only 4.2% (from 215 g/kWh to 224 g/kWh) at 1000 hours.

The combined system showed a 9–12% power loss (from 62 kW to 54.5 kW) at 300 hours, with fuel consumption increasing by 8.7% (from 222 g/kWh to 241 g/kWh). This degradation is driven by synergistic backpressure growth, which reduces both volumetric efficiency and turbocharger efficiency.

Table 4 presents the degradation mechanism comparison. The wet exhaust system demonstrates the fastest performance degradation due to scrubber-related blockages, while the dry system offers superior long-term stability. Exhaust backpressure and PM emissions are the primary indicators of degradation, with backpressure exceeding 18 kPa or PM emissions surpassing 0.4 g/kWh signaling critical failure thresholds. Thermal management in dry systems requires optimization to mitigate temperature-induced degradation, while wet systems need anti-sludge designs to prolong service life.

**Table 4:** Degradation mechanism comparison.

System Type	Primary Degradation Mechanism	Degradation Rate	Critical Failure Mode
Wet	Sludge accumulation + scrubber clogging + pipe corrosion	High (15–20%/500h)	Backpressure > 18 kPa; NOₓ approaching 4.70 g/kWh
Dry	Condenser coking + thermal fatigue cracking	Low (5–8%/1000h)	Condenser heat exchange efficiency decline; surface temperature > 135 °C
Combined	Dry-stage coking + wet-stage sludge accumulation	Medium (10–15%/300h)	PM > 0.4 g/kWh; unstable exhaust temperature

## 4. Failure mechanisms and modes analysis

1) **Wet Exhaust System:** Smoke baffle net deformation: Prolonged exposure to high-pressure exhaust gases (16–18 kPa) causes plastic deformation of the baffle net in the scrubber, reducing pore size by 30–40% after 500 hours. This leads to abrupt backpressure increases (e.g., from 16.23 kPa to 17.82 kPa at full load), as shown in Fig 14.

Pipe corrosion and leakage: Mixing of exhaust PM with coolant forms acidic sludge, causing pitting corrosion (depth≥0.5 mm) in carbon steel pipes. Leakage was observed at pipe joints after 500 hours, leading to coolant loss and reduced scrubbing efficiency.

2) **Dry Exhaust System:** Condenser tube thermal fatigue cracking: Cyclic temperature fluctuations (150–250°C) induce stress concentrations at welds, resulting in micro-cracks (length ≥1 mm). SEM analysis (Fig 15 cited image in [13,22] revealed transgranular cracking due to repeated thermal expansion/contraction.

schematic illustration of thermal fatigue cracking in dry system condenser tubes (theoretical model based on [13,22]). The proposed morphology is supported by FEA-predicted stress concentrations (≥180 kPa) exceeding the fatigue limit of SiMo iron (150 kPa) [13]. Note: The morphology aligns with literature-reported thermal fatigue patterns in high-SiMo alloys [13].

Nozzle blockage in exhaust dilution device: PM deposition (5–10 *μ*m particles) blocks 60% of nozzle pores after 600 hours, reducing dilution efficiency by 50% and increasing NOₓ emissions (Table 3). A hypothetical schematic (Fig 15) illustrates the thermal fatigue cracking mechanism in dry system condenser tubes:

**Remark 8**. Due to experimental limitations, SEM analysis was not conducted in this study, and we regret to note this omission. It should be noted that although SEM experiments were not performed, the exhaust systems for explosion-proof mine diesel engines are highly similar to those of conventional diesel engines. In fact, mine diesel engines operate under more complex conditions. Therefore, images from references [13,22] are cited in this paper to substantiate the failure mechanisms of exhaust systems for explosion-proof mine diesel engines.

## 5. Failure prediction models

To construct advanced failure prediction models, a hybrid approach integrating data-driven analysis and physics-based modeling is proposed, leveraging the paper’s experimental insights. First, a multivariable nonlinear regression model is developed to capture the coupled effects of operational parameters (rotational speed, load ratio) and environmental factors (ambient temperature, humidity) on exhaust backpressure degradation, as validated by the paper’s observation that wet systems exhibit 15.2% backpressure increase over 500 hours due to sludge accumulation. Second, physics-based models are formulated to describe thermal degradation in dry systems, incorporating heat transfer equations for condenser tubes and accounting for coking-induced efficiency decline (3–5% per 100 hours) observed in the paper. Third, machine learning algorithms (random forest) are employed to predict emission failures, using features like operational time, backpressure, and exhaust temperature, with thresholds derived from GB 20891−2014 limits (e.g., PM ≤ 0.4 g/kWh). Finally, a comprehensive failure index integrates these models, weighting parameters such as backpressure (≥18 kPa) and temperature (≥77°C) to enable proactive failure alarm based on the paper’s critical failure modes.

(1) Multivariable Nonlinear Backpressure Model [11,22,28]

Considering operational parameters (rotational speed *n*, load ratio *L*) and environmental factors (ambient temperature *T*_*a*_, humidity *H*, a nonlinear regression model for wet exhaust systems was developed:


$$\Delta P=3\times 10^{-4}t+9\times 10^{-5}n+2L+0.01T_{a}+0.5H+0.002tL+1\times 10^{-7}t^{2}$$ (1)

Where, *t* denotes the operating time (hour), *n* denotes the rotational speed (r/min), *L* denotes the load ratio, *T*_*a*_ denotes the ambient temperature (°C), *H* the humidity,

Validation: The model showed an $R^{2}=0.92$ against test data, outperforming the linear model. For example, at *t* = 5 (500 hours), *n* = 2200 r/min, *L* = 100%, *T*_*a*_ = 25 °C, *H* = 50%:


$$\begin{array}{c} \Delta P=3\times 10^{-4}\times 500+9\times 10^{-5}\times 2200+2\times 100\%+0.01\times 25+\\ \;\;\;\;\;\;0.5\times 50\%+0.002\times 500\times 100\%+1\times 10^{-7}\times 500^{2}=3.88kPa \end{array}$$ (2)

Consistent with the experimental value of 3.76 kPa.

**Remark 9**. The calculated result 3.88 kPa is not exactly consistent with the experimental value of 3.76 kPa. The difference arises from the inherent nature of the empirical regression model, which aims to capture the overall trend of backpressure increase rather than predict every data point with perfect accuracy. The discrepancy of 0.12 kPa represents a relative error of approximately 3.2%, which we consider to be well within the acceptable range for an empirical engineering model of this complexity, especially one that consolidates multiple operational parameters.

(2) Physics-based Thermal Degradation Model [13,24]

Incorporating heat transfer equations for dry systems:

Condenser heat transfer coefficient: *h* = 120-0.5*t*-0.01*T*_*g*_ + 0.02*v*. Where *T*_*g*_ denotes the exhaust gas temperature (°C), *v* denotes the gas velocity (m/s).

Exhaust temperature prediction: $T_{e}(t)=T_{e0}\exp\left(-\frac{hA}{mc_{p}}t\right)+T_{a}\left[1-\exp\left(-\frac{hA}{mc_{p}}t\right)\right]$ with *T*_*e*0_ denotes the initial exhaust temperature (°C), *A* denotes the heat transfer area (m²), *m* denotes the gas mass flow (kg/s), *c*_*p*_ denotes the specific heat (J/kg·K).

Parameter values: From Table 1 and Fig 4: *A* = 1.2 m², *m* = 0.15 kg/s, *c*_*p*_ = 1005 J/kg·K.

Example calculation: At *t* = 10 1000 hours, *T*_*e*0_ = 68 °C, *T*_*a*_ = 25°C, *h* = 85 W/m²·K:


$$T_{e}=68\exp\left(-\frac{85\times 1.2}{0.15\times 1005}\times 10\right)+25\left[1-\exp\left(-\frac{85\times 1.2}{0.15\times 1005}\times 10\right)\right]=72.3^{o}C$$ (3)

Which matches the experimental degradation trend.

(3) Machine Learning-based Emission Model [17,29]

A RF model was built for combined systems, using features: *t*, *n*, *L*, *T*_*e*_, Δ*P*, Historical emissions data (NOₓ, HC, CO, PM).

The RF model combines predictions from *M* = 100 decision trees, where each tree *f*_*m*_ maps input features *X*=[*t*, *n*, *L*, *T*_*e*_, Δ*P*, Historical Emissions] to a PM emission prediction:


$${\hat{y}}_{PM}=\frac{\text{1}}{M}\sum_{m=1}^{M}f_{m}(X)$$ (4)

Where ${\hat{y}}_{\text{PM}}$ is the predicted PM emission (g/kWh). Decision Tree Splitting Criterion: Each node in the decision trees is split to minimize Gini impurity, defined as:


$$Gini(D)=1-\sum_{k=1}^{K}{\left(\frac{\left|D_{k}\right|}{\left|D\right|}\right)}^{2}$$ (5)

Where *D* is the dataset at the node, *K* is the number of classes (for classification; adapted for regression via variance reduction), and *D*_*k*_ is the size of subset *k*.

The model structure includes: 1. 100 decision trees, 2. Gini impurity criterion. 3. Feature importance ranking: *t* (35%), *L* (25%), *T*_*e*_ (20%), Δ*P* (15%), n (5%).

PM emission prediction: $E_{PM}=f(t,L,T_{e},\Delta P,n)$

At *t* = 3 (300 hours), *L* = 75%, *T*_*e*_ = 67°C, Δ*P* = 18 kPa, *n* = 1400 r/min:

$E_{PM}=0.42g/kWh\;$ experimental: 0.40 g/kWh.

(4) Failure Threshold Integration [3,4,30,31]

A comprehensive failure index (FI) was defined: $FI\;=\alpha \frac{\Delta P}{P_{\lim}}+\beta \frac{T_{e}}{T_{\lim}}+\gamma \frac{E_{PM}}{E_{PM\lim}}$ with weight factors *α* = 0.4, *β* = 0.3, *γ* = 0.3, and limits *P*_lim_ = 18 kPa, *T*_lim_ = 77°C, *E*_PMlim_ = 0.4 g/kWh.

Failure criterion: FI ≥ 1

Example: Wet system at t = 500 hours:


$$FI\;=0.4\times \frac{18.7}{18}+0.3\times \frac{71.5}{77}+0.3\times \frac{0.35}{0.4}=1.02(critical\;failure)$$ (6)

Table 5 gives the four failure prediction models validation including the complexity, R², Error and the computational time. It can be seen that an increase in the number of parameters leads to more accurate model calculations. However, the application of neural networks like RF, while effectively reducing prediction error, also increases the required computation time. The R^2^ metric quantifies the association between variables and evaluates the model’s goodness-of-fit.

**Table 5:** Model validation and comparison.

Model Type	Complexity	R^2^	Prediction Error	Computational Time
Linear regression	Low	0.75–0.82	±15%	<1s
Multivariable NL	Medium	0.90–0.95	±8%	5–10s
Physics-based	High	0.88–0.93	±10%	15–20s
Random forest	High	0.94–0.97	±5%	20–30s

## 6. Conclusion

This study systematically investigates the long-term performance degradation and failure mechanisms of dry, wet, and combined exhaust systems for mining explosion-proof diesel engines, addressing critical gaps in short-term performance research ([6]). The key findings are:

1) The wet exhaust system exhibited the fastest degradation, with exhaust backpressure increasing by 15.2% within 500 hours due to sludge accumulation in the scrubber, leading to a 12–15% power loss. In contrast, the dry system showed 7.12–14.37% lower fuel consumption and 2.2–4.3% higher power output than the wet system over 1000 hours, though exhaust temperature increased by 3–4°C due to condenser coking.
2) Wet systems failed primarily from mechanical blockage (smoke baffle net deformation) and contamination, with critical failure occurring at 500 hours. Dry systems succumbed to thermal fatigue (condenser tube cracking) and coking, failing at 1000 hours. The combined system showed hybrid failures, with PM emissions exceeding GB 20891−2014 limits at 300 hours.
3) Dry systems reduce NOₓ, HC, CO, and PM emissions by 68.36%, 71.71%, 55.39%, and 82.28% respectively compared to wet systems, maintaining compliance with GB 20891−2014 over 1000 hours.
4) The developed multivariable nonlinear backpressure model (R² = 0.92), physics-based thermal degradation model (R² = 0.88–0.93), and RF emission model (R² = 0.94–0.97) accurately predict failure thresholds, enabling proactive maintenance.
5) Based on the study of this paper, we will focus on: 1) Creating a high-fidelity virtual digital-twin model of the exhaust system that updates in real-time with engine operating data to predict remaining useful life. 2) Moving from fixed-interval to condition-based maintenance, where alerts are generated automatically upon approaching predicted failure thresholds, thereby minimizing downtime and preventing catastrophic failures.

## Supporting information

S1 Data All Data. (XLSX)
